# A Qualitative Investigation Into What Parents Want From an Online Behavioural Sleep Intervention for Children With Epilepsy

**DOI:** 10.3389/fpsyg.2021.628605

**Published:** 2021-07-29

**Authors:** Georgia Cook, Paul Gringras, Harriet Hiscock, Deb K. Pal, Luci Wiggs

**Affiliations:** ^1^Centre for Psychological Research, Department of Psychology, Health and Professional Development, Faculty of Health and Life Sciences, Oxford Brookes University, Oxford, United Kingdom; ^2^Evelina London Children’s Hospital, London, United Kingdom; ^3^King’s College London, London, United Kingdom; ^4^Health Services Research Unit, Royal Children’s Hospital, Melbourne, VIC, Australia; ^5^Centre for Community Child Health, Murdoch Children’s Research Institute, Melbourne, VIC, Australia; ^6^Department of Paediatrics, The University of Melbourne, Melbourne, VIC, Australia; ^7^Department of Basic and Clinical Neuroscience, Institute of Psychiatry, Psychology and Neuroscience, King’s College London, London, United Kingdom; ^8^MRC Centre for Neurodevelopmental Disorders, King’s College London, London, United Kingdom; ^9^King’s College Hospital, London, United Kingdom

**Keywords:** qualitative, epilepsy, sleep, sleep intervention, parental needs, children

## Abstract

Many of the same sleep problems seen in typically developing (TD) children are frequently experienced by children with epilepsy (CWE). Behavioural sleep interventions (BSIs) are commonly and successfully used to treat these sleep problems in TD children and in some neurodevelopmental disorder populations. Therefore, BSIs should be effective in CWE, however, there are special seizure-related considerations for CWE and their parents which may be salient to consider in any future BSI development for this group. The current study sought to identify, from parents, if there were special considerations for the content and delivery of an online BSI for parents of CWE. Semi-structured interviews were conducted with nine mothers of CWE and thematic analysis was conducted on the interview data. Ten themes were apparent which represented what parents wanted from any online BSI for CWE. Parents wanted (i) other parents’ views and real-life experiences to be included, (ii) recognition of how changes over time may influence the appropriateness of using various sleep-management options, (iii) to be presented with a range of sleep management options from which they could select, (iv) personalised information and suggestions for behaviour-change options, (v) help to address child anxiety around sleep, (vi) for the advice and behaviour-change options to be practical, (vii) general educational information about sleep and the relationship between sleep and epilepsy, (viii) for parental worries and concerns to be acknowledged, (ix) to receive help, support, and reassurance around children’s sleep; and (x) to include the child in the intervention. It was clear that any online BSI would require specific adaptations and additions (to content and delivery format) to best meet the needs of parents of CWE. It is hoped that having identified what parents want from on online BSI for CWE will allow these factors to be acknowledged in future intervention development, with the intention to optimise parental engagement and intervention effectiveness. Practical suggestions for how these aspects could be integrated into any online BSI are suggested.

## Introduction

Epilepsy, characterised by recurrent seizures, is a common neurological condition which affects 1% of the population, with nearly 65,000 children and young people affected in the United Kingdom (Joint Epilepsy Council, 2011). Comorbidity is common in children with epilepsy (CWE) in domains such as cognitive functioning, memory, processing speed, and learning as well as behavioural problems (Children with Epilepsy in Sussex Schools (CHESS) study, 2014).

In various populations, a factor which has been linked with functioning in all these areas throughout the lifespan is sleep; poor-quality sleep in childhood predicts future cognitive, attentional, and psychosocial problems (Gregory et al., 2005; Hill et al., 2007; Simola et al., 2014). The relationship between sleep and seizure disorders is a particularly vicious cycle and readers are referred to the paper by Gibbon et al. (2019) for a consideration of the associations between sleep and epilepsy. Nocturnal seizures can fragment sleep, while a number of factors, including sleep disorders and anti-seizure medications, cause sleep fragmentation and can worsen seizures. Establishing and obtaining healthy sleep is particularly crucial in CWE as sleep disturbance (i.e., impaired quality or quantity of sleep) can also trigger seizures (Gibbon et al., 2019). Yet CWE experience sleep problems (i.e., symptoms suggestive of a possible sleep disorder) more frequently than typically developing (TD) children (Owens and Mindell, 2011), and this is true for children with epilepsy both with and without nocturnal seizures (Cottrell and Khan, 2005). For example, in a sample of 4–10 year old children with focal epilepsy, parental reported sleep problems were 12 times more common than in children without epilepsy, even without the presence of nocturnal seizures (Gutter et al., 2013).

The sleep problems experienced by CWE can lead to daytime sleepiness and worse cognitive functioning, behaviour, and quality of life (Stores et al., 1998; Maganti et al., 2006; Owens and Mindell, 2011). The impact extends to the whole family and parents of CWE often report having disturbed sleep (Larson et al., 2012). This may include disturbance due to waking up frequently to check on their child, or in some cases choosing to bed or room share with their child in case s/he has a seizure. Parents of CWE have been found to be at seven-times higher risk of sleep disturbance in comparison to parents of children without epilepsy (Shaki et al., 2011) and spend an average of only 4 h asleep, with further associated adverse outcomes on maternal health and marital satisfaction (Cottrell and Khan, 2005). Reducing sleep disturbance in CWE is therefore a pivotal target of intervention that could potentially improve not only child sleep, but also learning, mood, behaviour, seizures, and parental quality-of-life.

The type of sleep problems experienced by CWE are varied (see Gibbon et al., 2019 and Kothare and Kaleyias, 2010 for a detailed discussion of sleep issues in children with epilepsy) but can present as similar to the sleep problems experienced by TD children and commonly take the form of issues with sleep initiation (settling and going off to sleep) and/or maintenance (night or early morning waking) (Stores et al., 1998; Owens and Mindell, 2011; Gutter et al., 2013). These symptoms could arise as a result of various sleep disorders (or other factors related to the child’s epilepsy or other clinical conditions). Diagnosis and management decisions, of course, need to be based on careful individual assessment of each child and family. However, attention to behavioural factors (alone or as a component of intervention) is likely to form a part of management of many sleeplessness problems in both TD children and those with neurodevelopmental disorders (Wiggs and France, 2000; Mindell et al., 2006; Mindell and Meltzer, 2008; Bruni et al., 2018). Behavioural sleep interventions (BSIs), seek to provide parents with strategies they can implement to encourage desired sleep behaviours by manipulating their child’s learned associations with sleep. BSIs can be delivered in a variety of modes including face-to-face (Hiscock et al., 2015), telephone (Stuttard et al., 2015), paper-based (Gringras et al., 2012), and online/app based (Mindell et al., 2006; Espie et al., 2012), making for the possibility of flexible and cost-effective interventions with wide-reach. BSIs have well demonstrated efficacy in randomised controlled trials for younger TD children, and older (up to 12 years of age) autism and attention-deficit hyperactivity disorder (ADHD) populations (Mindell et al., 2006; Johnson et al., 2013; Hiscock et al., 2015). Therefore, it has been proposed that BSIs could be modified effectively for CWE (Gibbon et al., 2019).

However, a “one size fits all” approach to the behavioural management of sleep in CWE fails to acknowledge potential specific seizure-related considerations for CWE (e.g., nocturnal seizures or anxiety about seizures) and their parents (e.g., concerns about the appropriateness of using some behavioural techniques with a child who might have seizures). Qualitative approaches have increasingly been used to extend our understanding of key issues and experiences of parents and their CWE (Harden et al., 2016; Wo et al., 2018; Jones et al., 2019). Others have noted the extensive potential benefits of partnering with end users in the development of healthcare systems and interventions, including those to be administered online. This approach not only benefits the end product but can also engage and empower parents and families in their own healthcare and interventions (D’Alessandro and Dosa, 2001; Carman et al., 2013). While addressing sleep problems in CWE is a possible intervention target to improve a range of outcomes for children and their parents, there is a lack of exploration around what seizure-specific considerations or adaptations may be required for any BSIs for parents of CWE to best meet their needs. The current study was conducted as part of a larger program Changing Agendas on Sleep, Treatment and Learning in Epilepsy (CASTLE) exploring the health and quality of life of CWE and their parents, and whether these can be improved by better sleep. Epilepsy is a diverse group of electroclinical syndromes and how its various manifestations are linked with sleep and sleep difficulties at different ages could not, and was not intended to, be documented by the results of the current study. Rather, the current paper reports qualitative findings from interviews with parents of CWE that sought to identify broad factors related to sleep-management which were important to parents to inform the development and delivery of an online BSI designed for parents of CWE to be used in the CASTLE Sleep-E clinical trial^1^.

## Materials and Methods

### Participants and Recruitment

Nine mothers of children (six males, ages ranging 5–15 years, with median = 10 and mean = 10.3, SD = 2.9) participated in the initial interviews. The sample was generally well-educated. Descriptive information about parent-reported child sleep problems and seizure timing is shown in Table 1. Of the children, five had Benign Rolandic Epilepsy (one atypical), two had focal seizures, one generalised seizures, and one unspecified. Two had been diagnosed with epilepsy <1 year ago, two children between 1 and 3 years ago, and five children >3 years ago. As can be seen in Table 1, the children in this sample had experienced a range of sleep problems, either currently or in the past, with all children having difficulties with sleep currently and in the past. This allowed parents to provide their thoughts and experiences of dealing with longstanding sleep problems in their child with epilepsy.

**TABLE 1:** Descriptive information about children of the participants.

	Age	Gender	Seizure timing	Parent reported sleep problems*
P1	10	M	Transitioning between sleep and wake	Current: settling, night waking, sleep-related anxiety, unsettled sleep, co-sleeping
				Past: sleep terrors, sleep walking
P2	15	M	Daytime and during sleep	Current: settling, sleep-related anxiety, night and early morning waking, sleepwalking, sleep terrors, daytime sleepiness
				Past: room sharing
P3	10	F	Transitioning between sleep and wake	Current: night waking, sleep terrors, sleep-related anxiety, co-sleeping
				Past: morning waking difficulty
P4	11	M	Transitioning between sleep and wake	Current: settling, sleep-related anxiety, night waking
				Past: room sharing
P5	5	F	Daytime. 1 year without seizures (due to medication)	Current: settling, night waking, sleep terrors, sleep-related anxiety, room sharing, co-sleeping, daytime sleepiness
P6	9	M	During sleep	Current: settling, night and early morning waking
				Past: daytime sleepiness and co-sleeping
P7	13	F	During sleep and transitioning between sleep and wake	Current: settling, morning waking difficulty
P8	7	M	Transitioning between sleep and wake	Current: night and early morning waking, co-sleeping
P9	13	M	Daytime and transitioning between sleep and wake	Current: settling, night waking, poor sleep quality, daytime sleepiness, possible restless legs syndrome

*Current sleep problems refers to problems which are currently present but it should be noted that the duration of these problems is generally longstanding (since infancy or beyond), with most problems also being also present in the past. Past sleep problems refers to problems which have now resolved but which were significant problems at some point.

Participants were recruited (between March and July 2018) via online advertisements placed on the websites of epilepsy organisations and charities (e.g., Epilepsy Action) and the CASTLE study and researchers’ university websites. Online recruitment was considered appropriate given that taking part in the study required parents to have access to the internet.

Other inclusion criteria were that participants were the parent of a child with epilepsy (of any type), based in the United Kingdom and had sufficient English language skills so that they could read and interact with a draft version of the online BSI and respond to written and oral questions about it, reported in a separate paper (see Wiggs et al., 2021). There were no specific exclusion criteria and all parents who met the inclusion criteria were eligible.

Interested participants were invited to contact the researchers and once contact had been made, parents were emailed a participant information sheet explaining the study and a consent form. Potential participants were then contacted by the researchers to (i) ensure materials were received, (ii) discuss the specifics of the study and answer any parental questions, and (iii) complete an eligibility and contact details form if participants agreed to participate. Once signed consent forms were received (by post or scanned and sent via email), a convenient time was arranged to conduct the interview.

### Measures and Data Analysis

The initial intention had been to run focus groups with parents to elicit their thoughts and opinions about factors related to sleep-management which were important to them with the intention of using this information to inform the development of an online BSI for parents of CWE. However, logistical issues in bringing participants together at convenient times resulted in an amendment to the data collection method and individual semi-structured interviews were conducted with parent participants instead.

### Interviews

Researchers developed a semi-structured interview schedule that asked about key topics relevant to the development of an online sleep intervention (see Supplementary Material 1). In addition to asking about demographic factors and the child’s health and epilepsy for descriptive purposes, this included questions about (i) child and parental sleep (including asking about particular difficulties with sleep faced by the family and/or child because of the child’s epilepsy and the areas related to the child’s sleep that parents want help with), (ii) the nature and success of sleep-related treatments or management approaches that had been attempted (including their views about why some approaches they had tried were not successful, (iii) their views and experiences of the acceptability of behavioural interventions specifically, if not already discussed, (iv) epilepsy-specific issues that would need to be considered or addressed as part of any developed intervention, and (v) parental perceptions of what would make a good or bad online intervention experience (e.g., relating to website content and usability).

Whilst parents were asked about their own and their child’s particular experiences (sections i and ii), this was in the context of hoping to elicit broad issues related to sleep and sleep-management, rather than documenting their individual circumstances. Parents were fully informed at the outset about the intention of the interview and when answering encouraged, especially in sections (iii)–(v), to reflect on their own past and current experiences and the wider context of the parents of CWE community.

Interview transcripts were analysed by the researchers using thematic analysis following the standardised guidelines developed by Braun and Clarke (2006). The researchers further sought to ensure credibility, dependability and confirmability and applied a number of the “means for establishing trustworthiness” proposed by Nowell et al. (2017). An inductive data-driven thematic analysis approach was employed, whereby themes were derived from the data in a “bottom-up” approach, with participants’ words being the starting point from which themes were developed.

During data familiarisation, interviews were transcribed and then repeatedly reviewed, before being systematically coded by a researcher not involved in the interview process (GC). Coding was discussed amongst the research team to address any discrepancies and reach agreement in the coding. Because of these discussions, some codes were combined and other codes that did not relate to what parents of CWE wanted from any online BSI were omitted. One of the researchers (GC) then grouped the codes into potential themes. These themes were iteratively reviewed and discussed amongst the research team to reach agreement. Next, coded extracts of raw data were re-visited and the themes were reviewed across the whole data set to ensure that themes accurately reflected interview content. Subsequently, the research team agreed names and descriptions for each theme. This involved ongoing discussion until agreement was reached. Prior to the write-up, the research team agreed that the final themes reliably and accurately embodied the detail present in the participants’ original data. The final analysis was reviewed and discussed between the team until all were confident that the findings accurately represented the raw data and presented a coherent overview of the data relevant to the topic in question.

The presence of each theme across individual participants’ data was identified so that a frequency count for each theme could be generated to provide an indication of the strength of each theme across the sample. Results from the interviews allowed us to identify epilepsy-specific considerations for an online BSI for parents of CWE.

### Procedure

This was a qualitative study with parents of CWE. Ethical approval was obtained through the Oxford Brookes University’s Research Ethics Committee (UREC approval 171108). Following informed consent (as described in section “Participants and Recruitment”) interviews were scheduled and conducted (by LW and PG) at a time and in a manner convenient to participants (two face-to-face, six telephone, and one video call). They were audio recorded, transcribed and thematically analysed to identify the key themes reported by parents relevant to the development of the online intervention.

## Results

### Parent Interviews

Parent interviews had an average duration of 61 min. Several themes were apparent, as explained below along with supportive quotes, which highlighted key aspects parents felt should be acknowledged or addressed in any online BSI for parents of CWE. See Table 2 for an overview of final themes and which participants made comments relating to which theme. Details of the themes are presented in Table 3, including the theme description, frequency count and illustrative quotes.

**TABLE 2:** Summary of themes identified and by which participants they were reported.

	P1	P2	P3	P4	P5	P6	P7	P8	P9
Other parents’ views and experiences	x	x	x	x	x	x	x	x	
Change over time	x	x	x	x	x	x	x	x	
Range of management options	x	x	x	x	x		x	x	
Personalisation of information	x		x	x	x		x	x	
Child anxiety around sleep	x	x	x	x	x		x		
Practical sleep intervention suggestions		x	x			x	x	x	x
General sleep information		x	x	x		x	x	x	
Parental anxieties and concerns	x	x	X	x	x				x
Help, support and reassurance around sleep	x	x	x	x					
Include child in intervention		x				x	x		x

**TABLE 3:** Themes, description, frequency count and supportive quotes of what parents wanted to be included in an online BSI for parents of CWE (based on *n* = 9 parents).

Theme name	Theme description	Number of parents reported by	Example quotes
Other parents’ views and experiences	Access to other parents’ views or experiences (about parenting, sleep, interventions) were valued highly because other parents had first-hand experience and was seen as an essential aspect to include in the intervention	8	“I find it really helpful when you get stories from other parents who’ve tried things and if they’ve worked” (P4) “The real experiences from other parents” (P5) “I would more listen to other parents who would who have tried something, whether they had scientific research to back it up, if they said, ‘this has worked for my son’ and he’s got the same thing as (child) has, I think well it’s worth a go, I’ll give it a go” (P4) “It’s really nice to see or hear…or even read an excerpt from a parent” (P3)
Change over time	The need for any suggestions for behaviour change techniques to acknowledge that there are changes over time (in terms of the child’s epilepsy, age, the family’s anxieties) and that these affect the applicability of use of different techniques at different times	8	“Yeah, because it does massively change because I feel totally different to when he was first diagnosed. It was just none of us were getting any sleep at all. It was just really stressful” (P4) (in response to issues with putting things into practice) “I suppose it depends on…every child’s different aren’t they, and I think it probably just depends on your child and the age of your child” (P1) “That was the real crux of (child’s) changing and his sleep patterns was and that’s the year he really, really struggled was the change from primary school to secondary school, which apparently is meant to be one of the biggest things in a kid’s life anyway” (P2) “But what I would like to change, what I would maybe think about if and I’m thinking about it now because maybe the time will come and she will want to sleep alone, but maybe sleeping on her own would be something I would like to yeah try but not now with her epilepsy, no I wouldn’t give it a shot now” (P5)
Range of management options	That any suggestions for behavioural change techniques should be non-prescriptive but rather should give parents the ability to choose from a range of techniques, depending on what suited their individual child and/or circumstances	7	“if there was a website there to help you, to say ‘try this or try this or, you know, different things’… Because obviously different things work for different people. But you know if there was one kind of point you could go to get all of that advice that would have helped” (P4) “Here’s some things and see what fits in, what can you fit into your family, your circumstances, because the children will be at such a variety of ages” (P3) “If it’s an option then I would try to read through it” (P5) “So yes I think maybe almost like a spiders web isn’t it, so you have one and then it goes off and off and off. But you can still sort of come back in…:” (P2)
Personalisation of information	Parents had a desire to be directed to content which was personalised to the individual child and/or family needs as far as possible	6	“I think personalising it would make it more, have more of an impact. I mean at the end of the day the chances are if you’re reading about the intervention or being involved in the intervention then something going to resonate with you in that” (P1) “It would actually get me hooked up… to answer the data about my child and so that it pops up what kind of situation do we have so that it narrows down the data or narrows down the sort of text that I have to read…it would work for me” (P5) “Signposted to the most relevant part for yourself but then so if you [want], you know, this is available” (P3) “You also hope that by entering your problems…it’s the most interactive way to talk to the internet…you get some kind of answer” (P5)
Child anxiety around sleep	Many parents reported that their child struggled with anxieties around sleep. Either in relation to fear of sleep due to their epilepsy or as sleep being a time when anxieties or concerns were expressed. Some parents specifically desired information to help them support their child(ren) with any anxiety or fears around sleep	6	“He was having a lot of difficulty sleeping because he was worried that if he went to sleep, because we had quite a few instances where he went to bed as normal and when he woke up he was in hospital…he had a massive fear that he was going to die in his…sleep, so I think that’s, it’s not just the seizures, it’s the emotional side of it as well” (P2) “She does worry about things and it all seems to come out, you know, as it does I suppose you get into bed and you think about everything” (P7) “Maybe some sort of tool to talk to him about it” (P1) “It’s very hard and we’ve really tried but I know my husband has accidentally said it before. It’s very hard so if she’s resistant to want to go to bed he says ‘well (child) now look you know you need to go to bed early because you know because of your seizures you know’ and then you don’t want to bring it into that just before you go to bed” (P3)
Practical sleep intervention suggestions	Desire for practical suggestions for ways in which they could make changes to their child’s sleep	6	“I just want the end result of what to do…Get to the point, what do I need to do” (P7) “I think the main thing is, I think, to consider is it’s got to be something you can work around other family members” (P3) “So I think practical things as well help for reassurance for him and for us as well, yeah” (P2) “I think just practical stuff might be useful” (P6)
General sleep information	General sleep information (i.e., about normal sleep and the link between sleep and seizures) as well as specific “intervention” advice. Some parents had not been advised about the possible link between sleep and seizures while others were told by clinicians that good sleep was important for CWE but were not advised of ways to address child sleep	6	“Not anything that had ever been brought up or even asked…So no no one’s ever said anything about sleep” (P2) “I just Googled and Googled…for all different like helps and things like that to kind of help them” (P4) “I’ve done so much research online it’s ridiculous but it’s just like I’ve just sort of found out myself really that I try” (P7) “Sleep is the one thing that we can do, help. But then not really much assistance comes along with that at that point of diagnosis so it’s kind of, you know, you’ve got to be having 10 h, you’ve got to be getting them to sleep early and especially for children that are my daughters age, so 10/11, you’re talking quite a bit of a lifestyle change really for them” (P3)
Parental anxieties and concerns	For parents’ worries and concerns (about sleep, epilepsy and intervention approaches) to be acknowledged, even if these concerns could not all be resolved	6	“The problem for us is…that it’s us that are scared of leaving him in case he has a seizure so in regards to monitoring sleep I don’t really, you know, we’ve thought about putting a camera in his room and things but that would, we’d just be sat watching the camera” (P1) “….because you’re aware of these issues and, I think, as a parent to know that it’s been flagged up, that I’m not just being overreacting or being paranoid about letting my child go to a sleepover… Yeah, I think that would be nice, just to acknowledge it and say actually ‘no, there are other parents out there that either don’t let their child go to a sleepover”’ (P2) “I got so used to waking up and having to go into him, if he has a good night I become anxious and have to go through and check he is OK and still breathing” (P9) “I do sleep like a rabbit and I hear her every move and in her every move and every episode that she has during the night I am looking for signs that it might be a seizure” (P5)
Help, support and reassurance around sleep	That information about child sleep (typical and atypical), how to manage it and how this relates to others’ experiences would be reassuring and help parents feel supported. For some parents this was particularly needed at the time of diagnosis	4	“Having, yeah, reassurance I think, or even, yeah, just guidelines or something kind of like, this is what you can try for your child…” (P2) “It’s just so nice to know that there’s sort of someone out there who’s trying to do some research and trying to put things out there to help” (P1) “Because at the point of diagnosis they said this is, you know, the one thing you can do (sleep). It feels more pressured… more challenging, because you feel that it is the only thing you can do” (P3) “It was just really, really stressful [when first diagnosed] so any kind of assistance then would have been massive because I spent hours and hours on the internet like researching different things” (P4)
Include the child in intervention	Some parents felt that the child’s perspective, voice and/or feelings should be acknowledged in the intervention. It was seen as important that children’s perspectives about sleep had been considered and were included as they, and their needs, are central	4	“I think from a child’s point of view it’s very important too and maybe it would be even worth you interviewing some children about this because I don’t think they get their say about how they feel or how they would want to cope with it as well.” (P2) “I’ve noticed a lot of information that you get given is only aimed at the parents, if you get given anything, any kind of…leaflet or whatever this is for a parent of the child who has epilepsy and (child) is like, well, I’ve got it why am I not getting any information, why have they not given me anything to read…” (P6) “She is at an age now where yeah. But she, yeah, I think there should be information for both of us” (P7) “For the older children you should have some advice for them, the children as well so they can be part” (P9)

### Other Parents’ Views and Experiences

A firm preference was reported for hearing about other parents’ experiences, “*for me, when I’ve looked at websites and researched things, the thing that I look out for most is other parents’ stories*” (P1). Parents valued other parents’ experiences for a number of reasons; for some parents it was useful to hear what had worked successfully for other families, *“it is good to have other parents’ experiences as a backup, so if it doesn’t work maybe try this*” (P5). While for others, the value was in the sharing of experiences of parents dealing with sleep in CWE, *“as far as the sleep, not the medical side of sleep but the experience side, I think I would prefer to hear from other parents”* (P2).

For many parents, hearing from other parents was crucial and was perceived as more beneficial than simply being presented with information derived from clinical experience or research data:

*“So for me it’s definitely someone going through the same thing and reading their words is something that I would find maybe more powerful than just a paragraph saying, ‘well this normally happens or this can happen or that can happen”’* (P1).

Some parents specifically highlighted that an interactive component to any online resource would be useful in allowing them to obtain information and advice from other parents:

*“It’s a shame…you don’t have like a forum on it*…*so that parents can ask each other questions, that would be useful*…*because if there’s some random question that’s not answered on there*…*someone could go that’s happened to us this is what we did”* (P6).

Parents obviously valued hearing other parents’ views and experiences for a variety of different reasons and it was clear that this aspect would need to be a key component for any BSI designed for this group.

### Change Over Time

Parents reported a variety of possible ways in which they, their child and their broader family may change and develop over time, which were significant to managing their child’s sleep, and which needed to be acknowledged. These included changes in the child, the child’s epilepsy, age and family anxieties. Parents acknowledged that these factors may impact the relevance and/or use of different techniques at different times. For example, one parent emphasised individual child factors, and specifically age, as impacting the appropriateness of different behavioural interventions, *“I suppose it depends on*… *every child’s different aren’t they and I think it probably just depends on your child and the age of your child”* (P1). In addition, another acknowledged how the effectiveness of different sleep management techniques can shift and change over time, *“It helped for a while as things perhaps do”* (P7).

Parents stressed that variation could exist within a family across time. For example, one parent highlighted the difference age makes in parental monitoring and awareness of their child’s sleep and sleep problems, *“there’s a big difference between a 7 year old coming through going ‘mummy I can’t sleep’ then a 15-year old saying ‘oh I’ll sit on my iPad at 3 in the morning for 10 min until I fall back asleep again”’* (P2). Another was already anticipating possible future change, prompted by both her child’s wishes and her child’s seizure state:

*“But what I would like to change, what I would maybe think about if, and I’m thinking about it now because maybe the time will come and she will want to sleep alone, but maybe sleeping on her own would be something I would like to, yeah, try but not now with her epilepsy, no I wouldn’t give it a shot now”* (P5).

Parents clearly felt that any suggestions for managing children’s sleep needed to acknowledge the variety of possible changes which might occur over time and which may influence their desired sleeping arrangements, their approach to sleep as well as the applicability and/or use of different techniques at different time points.

### Range of Management Options

Parents felt strongly that they didn’t want sleep management options to be prescriptive, *“I don’t like anything*…*that sounds like this is the only way to go*…*it’s never black and white”* (P5). Instead, what parents desired and felt would have been useful to them when dealing with their CWE’s sleep was “*suggestions of things, ‘you could try this’, yeah and I think that would have been really helpful”* (P2).

Parents desired the ability to be informed about the range of different options, *“if there was a website there to help you, to say ‘try this or try this or, you know, different things”’* (P4). Parents felt that such information would allow them to decide which approach was most appropriate for them. For example, one parent said what they desired was to *“pick some that I felt could help and try those rather than try everything. You know try some that would fit in for us”* (P3).

Parents clearly valued the opportunity to review the range of possible sleep management options and to have the ability to choose techniques that they felt were most suited to them, their individual child and/or their individualised circumstances.

### Personalisation of Information

A key feature of any online BSI that parents felt was highly desirable was the personalisation of any material, where possible. For example, one parent identified the benefit of being signposted to key relevant information, *“you can be directed more specifically for your needs then that would be really good”* (P1). This desire for personalisation was emphasised, primarily for practical reasons so that parents could quickly identify information which was of relevance for them without having to spend time extracting this themselves. For example, one parent reported that:

*“I literally spent hours reading about different things, so if you could just do that then leave the bits that were relevant for you, definitely useful, yeah”* (P4).

Parents also felt personalisation would help material have the greatest benefit on parental engagement and how parents viewed the information, *“I think personalising it would make it more, have more of an impact”* (P1). It was clear that developing a means by which salient material could be identified and presented to parents in a personalised manner would be an important consideration for an online BSI for this group.

### Child Anxiety Around Sleep

It was common for parents of CWE to report that their children experienced anxiety around sleep as a result of their epilepsy, *“I’ve spoken to him about it and he said he used to be a bit nervous in case he did have one (seizure)”* (P4). For other parents sleep or bedtime was a time when child anxieties or concerns were explicitly expressed, “*bedtime is usually the time in which things come out if she is worrying”* (P3).

Some parents specifically reported that they were interested in finding out about ways to help them support their child(ren) with any anxiety or fears around sleep. For example, one parent reported what would be useful was *“how to explain and how to make an entry of this new subject would be helpful”* (P5). Others acknowledged that managing a CWE was not just about seizure management but also the anxieties and fears that are bound up in the condition for some children, *“it’s not just the seizures, it’s the emotional side of it as well”* (P2).

It was clear that CWE’s anxiety around sleep and bedtime was problematic and challenging for parents. Many parents also felt they required help and support to approach managing these issues suggesting this is an important component which should be included in any BSI for parents of CWE.

### Practical Sleep Intervention Suggestions

Parents reported a desire for approaches to and management options for their child’s sleep to be practical in nature. For example, when asked what would be useful one parent reported, “*I think just practical stuff might be useful”* (P6) and another “*just give people the practical stuff”* (P9).

Parents reported different motivations for desiring practical intervention suggestions including so that parents could easily identify the aspects of the intervention that they needed to implement, such as “*I just want the end result of what to do*…*Get to the point, what do I need to do” (P7).* While others wanted interventions that were practical for them and their individual circumstances:

*“I think the main thing is, I think to consider is it’s got to be something you can work around other family members’*” (P3).

Given parents’ desire for practical sleep management options, it appears important that any suggested management strategies are ones that can easily be integrated into family life and also that instructions for their use are conveyed to parents in a clear and easy to understand manner.

### General Sleep Information

It was evident that many parents did not feel that there was currently sufficient help and information for parents of CWE around sleep, *“we’ve not had any advice beyond that. Apart from get good sleep, that’s the one thing you can do”* (P3). Parents reported a desire for general information about sleep (including in relation to epilepsy):

*“*…*include the quality of the sleep so that if that is such a big factor in having seizures, I’d actually like to know more about sleep and how it can affect that”* (P3).

Some other parents also reported lacking awareness or knowledge of methods that they could use to manage any difficulties with sleep “*it’s a bit like I don’t really know what else there is really to do”* (P6). It was clear that many parents desired knowledge and information around sleep (i.e., normal sleep, relationships with epilepsy, sleep problems, and their management) and their need to feel informed should be addressed prominently in any BSI for parents of CWE.

### Parental Anxieties and Concerns

Parental anxiety was a topic which parents raised as salient and requiring acknowledgement. These anxieties were broad in nature and dealt with concerns about sleep, epilepsy and intervention approaches. For example:

*“We’re just scared, just the fear of it (seizure) happening and us not hearing him or us not being there is just, it’s just unbearable to think about”* (P1).

*“I got so used to waking up and having to go into him, if he has a good night I become anxious and have to go through and check he is OK and still breathing”* (P9).

Some parents acknowledged that the issues pertinent to them may not be able to be resolved by any intervention but it was nevertheless important that they were recognised:

“*…because you’re aware of these issues and I think as a parent to know that it’s been flagged up, that I’m not just being overreacting or being paranoid about letting my child go to a sleepover, yeah I think that would be nice just to acknowledge it”* (P2).

It is clear that parents’ wide-ranging worries and concerns about relevant topics need to be understood and acknowledged in a BSI for parents of CWE. In doing so, this will help provide parents with confidence that the intervention is sensitive to their needs and the challenges that they face.

### Help, Support and Reassurance Around Sleep

Many parents reported not feeling adequately supported in managing and, if necessary, improving CWE’s sleep. For example, “*I think any kind of help, kind of assistance with getting to sleep….because a lot of them, I think, do struggle to sleep”* (P4). Many parents felt that it would be reassuring and help parents feel supported if they had more access to information about child sleep (both typical and atypical), how to manage it and also how this information and their own experiences relate to others’ experiences:

*“Having, yeah, reassurance I think, or even, yeah, just guidelines or something kind of like, this is what you can try for your child*…*”* (P2).

For some parents it was clear that this type of help, support and reassurance was particularly pertinent at the time of diagnosis:

*“Because at the point of diagnosis they said this is, you know, the one thing you can do (sleep). It feels more pressured*… *more challenging, because you feel that it is the only thing you can do”* (P3).

It was evident that support and reassurance for parents around managing and, if necessary, treating their child’s sleep was lacking. For some parents, this was a fundamental and essential element that should feature in any BSI developed for this group.

### Include Child in Intervention

Some parents felt that CWE’s perspective, voice and/or feelings should be clearly acknowledged in the intervention:

*“I’ve noticed a lot of information that you get given is only aimed at the parents, if you get given anything, any kind of leaflet or whatever this is for a parent of the child who has epilepsy and (child) is like, well, I’ve got it why am I not getting any information, why have they not given me anything to read*…*”* (P6).

Specifically, it was felt by some that children’s perspectives about sleep should be considered and included as they, and their needs, are central and this is not an aspect which is usually addressed:

“*I know with (child) nobody’s ever asked him how he feels and that’s one thing he keeps on about quite a lot. That nobody understands how he feels, which we don’t”* (P2).

Some parents highlighted specific means by which children’s perspectives could be integrated and how children could be involved in different ways in the intervention:

*“I think from a child’s point of view it’s very important to and maybe it would be even worth you interviewing some children about this because I don’t think they get their say about how they feel or how they would want to cope with it as well”* (P2).

It appears important and relevant that an online BSI designed for parents also acknowledges or includes the voice of CWE themselves.

## Discussion

Ten themes were identified which represented the requirements of parents of CWE for any online BSI. Parents wanted (i) other parents’ views and real-life experiences to be included; (ii) recognition of how changes over time may influence the appropriateness of using various sleep-management options; (iii) to be presented with a range of sleep management options from which they could select; (iv) personalised information and suggestions for behaviour-change options; (v) help to address child anxiety around sleep; (vi) for the advice and behaviour change options to be practical; (vii) general educational information about sleep and the relationship between sleep and epilepsy; (viii) for parental worries and concerns to be acknowledged; (ix) to receive help, support, and reassurance around children’s sleep; and (x) to include the child in the intervention.

Sleep issues (including broader sleep-related factors which might not indicate any sleep disturbance, a sleep problem or a sleep disorder, for example to do with the sleep environment or parent anxiety) are common in CWE and parents in the current study were very clear about the need for a sleep resource. This desire for information and support is in keeping with the findings of another qualitative study with parents of CWE, which also emphasised parents’ desire for both information *and* support, which extends beyond the point of diagnosis (Jones et al., 2019). Many parents felt that receiving information and support around child sleep would help them to feel reassured about their ability to manage and, if necessary, improve their child’s sleep. The current study has described the key aspects that parents of CWE would want to be included in any online BSI for this group and it is hoped developing an online BSI with these considerations in mind would help to meet this need.

One of the most prominent topics reported by parents was the importance of hearing from other parents who have shared experiences. This desire has also been highlighted as an important factor for parents of CWE in a Malaysian sample (Wo et al., 2018). Based on the frequency and strength with which this theme was reported by parents in the current study it was concluded this was an essential addition to any online BSI for parents of CWE. A possible practical approach, which is increasingly recognised for its range of benefits in health research, is to involve parents in the co-production of the intervention (Hickey et al., 2018). In addition, explicitly representing the views and experiences of actual parents of CWE as part of the intervention content, perhaps in the form of videos or quotations, could also be beneficial and achievable as a way to clearly present other parents’ experiences and help foster engagement (D’Alessandro and Dosa, 2001; Carman et al., 2013). Some parents in the current study expressed that they valued the use of interactive sites such as parent forums for this reason, so that they could exchange information, techniques or to obtain answers to relevant questions that they had. Peer support has been identified as a key factor in contributing to self-management in a young adult with narcolepsy (Franceschini et al., 2020), increasing feelings of social support and fostering information sharing. These benefits of peer support are similar to those outlined by the mothers in the current study, when describing the advantages associated with the peer support they received via online parenting forums. However, there may be tensions between combining anecdotal information from forums with evidence-based information included in any online BSI.

Another theme highlighted by parents was the possibility of including or representing CWE themselves in any online BSI. The nature of any sleep issues and the age range of the children targeted by the intervention would likely influence whether this was possible and the best approach for achieving this. This could perhaps, as suggested by some parents in the current study, take the format of having age-appropriate accompanying materials/online resources designed for the child which may further contribute to a feeling of partnership in managing children’s sleep. A qualitative study of a young adult with narcolepsy has highlighted how peer support can improve an individuals’ self-management of their condition (Franceschini et al., 2020). Perhaps an intervention approach that fostered peer support between CWE would be beneficial in the management of the child’s condition and/or sleep, offering them an additional opportunity for support. Some parents in the current study also suggested having children’s input into the intervention content, however, the appropriateness of this may be limited, particularly as some BSI strategies are specifically designed to eliminate parent behaviours which the child themselves may find rewarding and have little motivation to have eliminated. Involving CWE in the form of interviews and reflecting their voice and key concerns and experiences throughout the intervention would be another, perhaps more achievable, way in which children could be “included”. Identifying how best to offer peer support to CWE and their parents is an important area for future investigation.

Parents were supportive of a diverse range of different behavioural techniques being provided (including those which they may not choose to use themselves) to address many types of sleep problems. The possibility for personalisation and freedom in the choice of which techniques to use was also important as it allowed parents to select strategies which were relevant, practical, and acceptable for them and their family at any given time. Including parents of CWE in decision-making has been highlighted as a key aspect of paediatric epilepsy care (Berg et al., 2013). Offering a range of possible intervention choices for dealing with each sleep problem and ensuring that these are presented as options rather than “instructions,” via sensitive phrasing, could be a helpful way to best meet parents’ needs.

Parents valued personalisation because it acknowledged the uniqueness of their child and family and also for practical reasons, as it limited the amount of information they would have to attend to (if they wanted to be more selective in their reading). The value of adopting a customised approach when delivering sleep interventions has also been raised by a sample of parents of disabled children (Beresford et al., 2010). The feeling of being overwhelmed by the excess of, often irrelevant, information generated from an internet search was prominently expressed by parents in the current study and in previous studies which have explored internet use in parental information seeking about child health care more broadly (Walsh et al., 2015). Ensuring any online BSI for parents of CWE was personalised may therefore help to encourage parental engagement with the material for multiple reasons.

Parents made it clear that any sleep management options should be practical. The current study explored what parents wanted from an online BSI and so, by definition, the strategies that would be presented would be behavioural in nature. These types of techniques are underpinned by the idea that parents follow clearly described strategies which are designed to be practical for parents to implement. However, additional attention to how these strategies are presented (e.g., noting possible variations which could be used, advice for coping with common situations which could arise during intervention) may help to ensure they are explicitly perceived by parents as “practical” strategies.

It was clear that parents of CWE held broad concerns and anxieties relating to their child’s sleep, epilepsy and use of intervention approaches. Many parents felt that the pervasive and ongoing nature of these anxieties needed to be acknowledged in any credible BSI. Therefore, it is essential that any BSI comprehensively considers and seeks to allay, if possible, common parental anxieties. In some cases, it may be sufficient to reassure parents by providing them with evidence-based information. In others, the sensitivity with which matters are presented and discussed may be relevant. However, even where no obvious resolution to their anxieties is possible, parents believed it was important for the possible presence of such concerns to be aired and recognised, to increase the credibility of the BSI.

Parents also felt that content of any online BSI needed to acknowledge specific changes that may occur over time in the lives of parents and CWE. In the current study, these changes most notably included the status of the child’s epilepsy, the child’s age and the family’s anxieties. Parents explicitly stated that changes in these areas could affect how they felt about any online BSI and also their views about the applicability of using different behavioural techniques at different times. Offering parents a range of optional techniques from which they can select goes some way to addressing this. In addition, material could explicitly make reference to the importance of these child and family factors, for example by ensuring that there is coverage of any sleep-related developmental changes and discussion of pertinent child and family factors which might influence parental choices about use of specific intervention strategies.

Many parents desired knowledge and information around sleep. This is in line with previous studies which found mothers of CWE in a Taiwanese cohort had relatively poor knowledge of child sleep (Tsai et al., 2018). Therefore, a key component of a BSI developed for this group should be the provision of general information regarding sleep. In the current study parents reported specifically wanting general sleep information about normal sleep processes, the links between sleep and seizures and details of specific methods that they could use to manage any difficulties with sleep in CWE. While limited, there is evidence to suggest that reduced levels of maternal knowledge about child sleep is linked to poorer and more variable maternally reported sleep in CWE (Tsai et al., 2018). Although there may be cultural influences on this reported link between maternal knowledge and reporting about sleep and these findings need further exploration, such results suggest that ensuring adequate levels of parental knowledge about sleep may be an important intervention target, helping parents to understand their child’s sleep processes or to feel more confident and informed about how to manage their child’s sleep.

Previous research has highlighted that CWE experience higher levels of anxiety around sleep in comparison to controls (Stores et al., 1998). It was apparent from the current study that a specific aspect of CWEs’ sleep for which parents desired further support was managing sleep-related anxiety and night-time fears, which could play a role in many sleeplessness problems of CWE. Therefore, any future BSI for this group should ensure this topic is explicitly addressed so that the content reflects the type of sleep difficulties which are prominent for CWE.

A potential limitation of the current study is the small sample size, particularly as “CWE” are heterogeneous. Whilst broad and varied recruitment strategies were employed, initially for focus groups and later for interviews, recruitment remained a challenge throughout. The final sample consisted of only nine parents, all of which were mothers who were generally well-educated. However, these were mothers of CWE aged 5–15 years and this allowed us to obtain perspectives based on their experiences across childhood as these parents had dealt with a broad range of longstanding sleep-related difficulties. There generally seemed to be considerable similarity across parents in terms of their views about the priorities for factors which should be included in any online BSI for CWE with most of the themes, endorsed by the majority of parents. Greater understanding of demographic factors related to parents’ views (and eventual use) of online interventions would be helpful. Further, the current study did not attempt to relate parents’ views to the particular problems with sleep or sleep-management identified by the families as these could never be representative of “CWE.” Instead the intention was to highlight participants’ opinions about general topics which are relevant for families, so that standardised BSIs could be developed and presented with these considerations in mind. Whilst some elements of the findings are clearly specific to parents of CWE (e.g., wanting information about the links between sleep and seizures), we don’t know if other themes related to the delivery of an online sleep intervention (e.g., a desire for other parents’ views and real-life experiences to be included and recognition of how changes over time may influence the appropriateness of using various sleep-management options) are specific to parents of CWE or are also important to other groups of parents or clinical groups. Future studies exploring the needs of parents in relation to child sleep interventions for discrete clinical (and other) groups are needed and could helpfully expand the number of parents involved and include fathers and children to maximise the chances of capturing diverse perspectives.

Being provided with information about management of behavioural sleep disorders will not be appropriate or the entire sleep-solution required for all parents of CWE. However, attention to behavioural factors (alone or as a component of intervention) is likely to form a part of the management of many sleeplessness problems, including in CWE. The availability of a general, online BSI resource for all parents of CWE would therefore seem useful. Whilst behavioural interventions for child sleep problems have a strong evidence base, parental insights from the current study suggest that the way management advice is delivered to parents could affect their engagement and, in addition, for parents of CWE, there are areas of additional content which should also be addressed to best meet their particular needs. The development of an online BSI (Castle Online Sleep Intervention, COSI) for parents of CWE which attempts to address the points raised by parents in the current study, is reported elsewhere (Wiggs et al., 2021) and its efficacy will be evaluated in the forthcoming CASTLE Sleep-E clinical trial (see text footnote 1). It is hoped that taking account of the special considerations of BSIs for parents of CWE will maximise the chances that the intervention is perceived as being relevant for them and their family.

## Data Availability Statement

The datasets presented in this article are not readily available because the data are qualitative interviews and cannot be shared. Requests to access the datasets should be directed to Amber Collingwood, amber.collingwood@kcl.ac.uk.

## Ethics Statement

The studies involving human participants were reviewed and approved by the Oxford Brookes University Research Ethics Committee (UREC approval 171108). The patients/participants provided their written informed consent to participate in this study.

## Author Contributions

All authors contributed to conception and design, acquisition of data, analysis and interpretation of data, and drafting the article or revising it critically for important intellectual content.

## Conflict of Interest

The authors declare that the research was conducted in the absence of any commercial or financial relationships that could be construed as a potential conflict of interest.

## Publisher’s Note

All claims expressed in this article are solely those of the authors and do not necessarily represent those of their affiliated organizations, or those of the publisher, the editors and the reviewers. Any product that may be evaluated in this article, or claim that may be made by its manufacturer, is not guaranteed or endorsed by the publisher.

## Footnotes

1
https://castlestudy.org.uk/
